# Introducing and Implementing HIV Self-Testing in Côte d'Ivoire, Mali, and Senegal: What Can We Learn From ATLAS Project Activity Reports in the Context of the COVID-19 Crisis?

**DOI:** 10.3389/fpubh.2021.653565

**Published:** 2021-07-20

**Authors:** Arsène Kouassi Kra, Géraldine Colin, Papa Moussa Diop, Arlette Simo Fotso, Nicolas Rouveau, Kouakou Kouamé Hervé, Olivier Geoffroy, Bakary Diallo, Odé Kanku Kabemba, Baidy Dieng, Sanata Diallo, Anthony Vautier, Joseph Larmarange

**Affiliations:** ^1^Centre Population et Développement (Ceped), Institut de Recherche pour le Développement (IRD), Université de Paris, Inserm, Paris, France; ^2^Solidarité Thérapeutique et Initiatives pour la Santé (Solthis), Dakar, Senegal; ^3^Solidarité Thérapeutique et Initiatives pour la Santé (Solthis), Abidjan, Côte d'Ivoire; ^4^Solidarité Thérapeutique et Initiatives pour la Santé (Solthis), Bamako, Mali

**Keywords:** HIV self-testing, COVID-19, West Africa, Côte d'Ivoire, Mali, Senegal, key populations

## Abstract

**Background:** The ATLAS program promotes and implements HIVST in Côte d'Ivoire, Mali, and Senegal. Priority groups include members of key populations—female sex workers (FSW), men having sex with men (MSM), and people who use drugs (PWUD)—and their partners and relatives. HIVST distribution activities, which began in mid-2019, were impacted in early 2020 by the COVID-19 pandemic.

**Methods:** This article, focusing only on outreach activities among key populations, analyzes quantitative, and qualitative program data collected during implementation to examine temporal trends in HIVST distribution and their evolution in the context of the COVID-19 health crisis. Specifically, we investigated the impact on, the adaptation of and the disruption of field activities.

**Results:** In all three countries, the pre-COVID-19 period was marked by a gradual increase in HIVST distribution. The period corresponding to the initial emergency response (March-May 2020) witnessed an important disruption of activities: a total suspension in Senegal, a significant decline in Côte d'Ivoire, and a less pronounced decrease in Mali. Secondary distribution was also negatively impacted. Peer educators showed resilience and adapted by relocating from public to private areas, reducing group sizes, moving night activities to the daytime, increasing the use of social networks, integrating hygiene measures, and promoting assisted HIVST as an alternative to conventional rapid testing. From June 2020 onward, with the routine management of the COVID-19 pandemic, a catch-up phenomenon was observed with the resumption of activities in Senegal, the opening of new distribution sites, a rebound in the number of distributed HIVST kits, a resurgence in larger group activities, and a rebound in the average number of distributed HIVST kits per primary contact.

**Conclusions:** Although imperfect, the program data provide useful information to describe changes in the implementation of HIVST outreach activities over time. The impact of the COVID-19 pandemic on HIVST distribution among key populations was visible in the monthly activity reports. Focus groups and individual interviews allowed us to document the adaptations made by peer educators, with variations across countries and populations. These adaptations demonstrate the resilience and learning capacities of peer educators and key populations.

## Introduction

HIV testing is an essential part of the epidemic response. It allows undiagnosed people living with HIV (PLHIV) to be linked to care and antiretroviral treatment and those testing negative to be linked to appropriate HIV prevention services (1).

HIV self-testing (HIVST) is a process in which users collect a sample (oral fluid or blood) themselves, perform the HIV test, and then interpret the result alone, often in a private setting (2). It is an innovative tool that empowers individuals and ensures the confidentiality of the test (3). Since 2016, the World Health Organization (WHO) has recommended HIVST as an additional approach to HIV testing (4).

In Southern and Eastern Africa, HIVST has begun to be massively deployed, notably through the Unitaid-funded STAR–HIV Self-testing Africa Initiative, initiated in 2015 (5). Previous studies have suggested that, for many users, HIVST promotes discretion and autonomy, and greatly increases the use of testing (6–8). HIVST is highly acceptable, particularly among key populations and those who do not regularly test for HIV. Initial feedback shows the acceptability, feasibility, and excellent clinical performance of HIVST (9–14). HIVST does not reinforce risk behaviors; on the contrary, it can increase condom use, e.g., among female sex workers (14), and positively impacts health behaviors (15, 16). Finally, some studies have shown that HIVST does not increase negative social consequences or undesirable events or behaviors (17).

Until 2019, access to HIVST remained low in West and Central Africa and was mainly limited to pilot programs (18). Uptake of HIV testing in this region is generally low: in 2019, only 68% (compared to 87% in Eastern and Southern Africa) of PLHIV were aware of their HIV status. According to UNAIDS, in 2019, only 81% of PLHIV knew their HIV status (19).

West Africa is characterized by mixed HIV epidemics: national HIV prevalence rates in the adult population are lower than in southern Africa (between 0.4 and 3%), but HIV remains widespread, and high prevalence rates (>10%) are observed in key populations (female sex workers—FSW, men who have sex with men—MSM, and people who use drugs—PWUD).

Funded by Unitaid and coordinated by Solthis, the ATLAS program *(AutoTest VIH, Libre d'Accéder à la connaissance de son Statut)* aims to promote and implement HIVST in Côte d'Ivoire, Mali and Senegal. This involves distributing nearly half a million HIVST kits as part of the three countries' national AIDS strategies and the integration of HIVST with the testing policies already in place. The different delivery channels and priority populations for each country were developed with country stakeholders (national AIDS programs/councils, international institutions including the WHO, international and national non-governmental organizations—NGOs—involved in local HIV programs, and civil society and community leaders).

ATLAS HIVST distribution is organized through eight different operational delivery channels (Supplementary Figure 1): five are facility-based (delivery of HIVST kits through public or community-based health facilities), and three use a community-based approach involving outreach activities engaging FSW, MSM, and PWUD (20). Peer educators conduct these outreach activities through group activities (e.g., talks, discussion groups, night visits, social events, parties) and face-to-face activities (e.g., home visits). Outreach activities represent the majority (more than two-thirds) of ATLAS's delivery objectives. HIVST distribution targets were fixed with implementing partners based on their past experiences and capacities. Therefore, the volume of HIVST kits distributed per channel is not exactly proportional to the weight of each population within the local HIV epidemics.

ATLAS activities rely both on primary distribution—HIVST kits are distributed by peer educators and healthcare professionals to primary contacts for their personal use—and secondary distribution—primary contacts are invited to redistribute some HIVST kits to their peers, partners, and relatives. These secondary contacts are often members of key populations that are more difficult to engage in HIV prevention, along with other peripheral vulnerable populations. This specificity of HIVST implies that HIVST beneficiaries (end users) are not limited to primary contacts. To preserve the anonymity and confidentiality of HIVST and not impede the use of HIVST, ATLAS decided not to track systematically distributed HIVST kits, which could be counterproductive. However, HIVST users can, if they wish, obtain additional support by calling a peer educator or the national HIV hotline.

HIVST distribution started in mid-2019 but was soon impacted by the COVID-19 pandemic (21). In response to the health emergency, the governments of Côte d'Ivoire, Mali, and Senegal, like those of other countries, adopted various public health measures (physical distancing in public spaces, protective masks, hygiene measures) (22). Other more restrictive measures, such as restrictions on international and domestic travel, curfews, and the closure of party venues and shops, were also adopted, making it difficult to carry out the ATLAS activities as initially planned.

Aware of these issues, Solthis and its implementing partners have had to adapt their field activities to each local context and each delivery channel; the operational challenges are significantly different between channels using facility-based and those using community-based strategies.

This article will focus solely on community-based outreach strategies, considering the set of unique challenges faced by peer educators. We will refer to them as FSW-based, MSM-based, and PWUD-based channels, considering the type of key populations targeted as primary contacts, and keeping in mind that secondary contacts are not systematically from the same key population.

From the program data (both quantitative and qualitative) collected by the ATLAS program, we examine temporal trends in the community-based distribution of HIVST and describe their evolution in the context of the COVID-19 health crisis. Specifically, we investigate the impact on, the adaptation of, and the disruption of field activities. What adaptations have been made by HIVST distributors? How did they integrate COVID-19 hygiene measures? What remained after the easing of governmental measures?

## Materials and Methods

### Sources of Data

We conducted a secondary analysis of the program data collected in the context of the monitoring and evaluation component of ATLAS: (i) quantitative monitoring data corresponding to the monthly activity reports of the various implementing partners; (ii) focus groups routinely conducted with HIVST distributor agents organized annually as part of monitoring and evaluation to collect qualitative feedback; and (iii) *ad hoc* individual interviews conducted by Solthis with peer educators during the Covid-19 pandemic specifically to document activities' adaptations in this specific context.

#### Monthly Activity Reports

All ATLAS implementing partners (public sector and civil society organizations—CSOs) provide monthly activity reports collected through a web platform specific to the ATLAS program and based on DHIS2 software (https://www.dhis2.org/). For the three community-based delivery channels, the monthly reports include, per channel (i.e., FSW-based, MSM-based, PWUD-based) and per intervention site: the number of interventions (or activities) conducted during the month, the number of primary contacts seen during interventions and who received one or more HIVST, and the number of distributed HIVST.

Primary contacts can be disaggregated by sex and age group (24 or under, 25–49, and 50 and over). Activities are also disaggregated by type (e.g., focus groups, home visits…). In addition, the distribution objectives, set upstream by Solthis with its implementing partners, have also been entered on the monitoring-evaluation platform by month, channel, and country.

#### Focus Group Discussions

ATLAS's monitoring and evaluation routinely include gathering qualitative feedback from the field through focus groups conducted regularly with distributor agents from each country and each delivery channel. These focus groups are led by different facilitators trained in conducting qualitative interviews.

Two waves of focus groups have been conducted: the first from October to November 2019 and the second in October 2020. Focus group participants were invited by ATLAS country operational teams in collaboration with their structures/organizations. Indications were given to ATLAS country operation teams to diversify the origin of participants (region, organizations…). It was not the same participants in 2019 and 2020.

All the focus groups were conducted face to face, with appropriate hygiene measures and physical distancing for those held in October 2020. While in 2019 the discussion topics mainly addressed the initiation of activities, operational challenges, and primary contacts' perceptions of HIVST, the focus groups conducted in 2020 included COVID-19-related issues and the resulting adaptations. For this article, only the group interviews conducted in 2020 with HIVST distributors involved in community-based outreach strategies were taken into account, i.e., 3 focus groups for Côte d'Ivoire, 2 for Mali, and 3 for Senegal (in Mali, no activities are targeting PWUD). The focus groups were audio-recorded with the agreement of the participants. At the beginning of the group interviews, participants were reminded of the confidentiality rules. Each participant was given a number to refer to each other without using their names. The focus groups were transcribed by the facilitator who conducted the focus group and then coded (with any personal identifiers removed).

#### Individual Interviews

Furthermore, because of the particular health context linked to the COVID-19 pandemic, Solthis wanted to set up a specific monitoring system to understand the adaptations implemented by field workers and guide program recommendations. Additional semi-structured individual interviews were carried out by telephone between September 8 and October 19, 2020, with peer educators distributing HIVST kits to key populations. Fourteen individual interviews were conducted by the second author (6 women and 8 men; 4 interviews in Côte d'Ivoire, 4 in Mali, and 6 in Senegal). The individual interviews were audio-recorded with the agreement of the participants, transcribed by the second author, and then coded (with any personal identifiers removed).

### Data Analyses

#### Quantitative Analyses

The temporal trends of the different quantitative indicators are presented here by month and stratified by country and delivery channel, taking into account monthly reports between August 2019 (initiation of activities) and December 2020.

Activities are reported by type in the monthly reports. However, the terminology used for activity type varies by country, channel, and implementing partner, making comparisons difficult. Instead, as the number of primary contacts and the number of activities are reported for each type (per month, site, delivery channel, and implementing partner), we calculated for each line of the monthly reports an average number of primary contacts per activity and thus categorized the activities into five groups according to this average number of contacts per activity (cpa): activities conducted face-to-face (cpa ≤ 1. 5), in small groups of 2–4 people (1.5 < cpa ≤ 4.5), in medium groups of 5–7 people (4.5 < cpa ≤ 7.5), in large groups of 8–10 people (7.5 < cpa ≤ 10.5) and in very large groups of 11 or more people (cpa > 10.5).

For metrics corresponding to ratios (e.g., the average number of distributed HIVST kits per primary contact or the average number of primary contacts per activity), 95% confidence intervals were calculated assuming a Poisson distribution.

#### Qualitative Analyses

The individual interviews conducted by the second author and the focus groups conducted by trained facilitators were initially not designed for scientific qualitative analysis but rather as part of the operational evaluation of the activities.

The second author performed the coding of the individual interviews based on an initial content analysis to identify emerging themes and produce an operational guide of good practices regarding HIVST activities in the context of Covid-19 (available on https://atlas.solthis.org/wp-content/uploads/2021/02/Adaptation-ATLAS_COVID.pdf).

For this paper, the transcriptions of individual interviews and focus groups were reanalyzed together by the second author to describe how HIVST activities targeting key populations were adapted in response to the COVID-19 crisis and identifying convergences and divergences between countries and delivery channels. The themes and subthemes were updated based on discussions between the two first and the two last authors. Verbatims were selected to illustrate the different subthemes retained for the paper.

### Ethical Authorizations

Secondary analysis of ATLAS program data is included in the associated research protocol available at https://atlas.solthis.org/en/research/. This protocol (version 2.1, August 5, 2019) has been approved by the WHO Ethical Research Committee (August 7, 2019, reference: ERC 0003181), the National Ethics Committee for Life Sciences and Health of Côte d'Ivoire (May 28, 2019, reference: 049-19/MSHP/CNESVS-kp), the Ethics Committee of the Faculty of Medicine and Pharmacy of the University of Bamako, Mali (August 14, 2019, reference: 2019/88/CE/FMPOS), and the National Ethics Committee for Health Research of Senegal (July 26, 2019, protocol SEN19/32).

### Context: Governmental Health Measures in Response to COVID-19

Following the first wave of the COVID-19 pandemic in early 2020, the governments of Côte d'Ivoire, Mali, and Senegal implemented health measures in mid-March 2020 (Table 1). Group gatherings were banned from March 15 in Senegal, March 16 in Côte d'Ivoire, and March 19 in Mali. In all three countries, a state of health emergency was declared (on March 20 in Mali and on March 23 in Côte d'Ivoire and Senegal), followed by curfews (on March 23 in Senegal, March 24 in Côte d'Ivoire, and March 26 in Mali) and other measures restricting movement (for example, restrictions on movement between regions or between the capital and other regions). While Europe and North America were particularly affected during this first wave, the number of cases recorded in West Africa has remained limited (23).

**Table 1:** Main health measures implemented during the COVID-19 crisis in 2020 in Côte d'Ivoire, Mali, and Senegal.

**Month**	**Day**	**Côte d'Ivoire**	**Mali**	**Senegal**
March	15			Ban on public gatherings Closure of restaurants, bars, nightclubs, and entertainment venues
	16	Ban on public gatherings		
	18	Closure of restaurants, bars, nightclubs, and entertainment venues		
	19		Ban on public gatherings Closure of bars and nightclubs	
	20		Public Health Emergency Declaration	
	23	Public Health Emergency Declaration		Public Health Emergency Declaration Curfew Limited travel between regions
	24	Curfew		
	26	Limited travel between Abidjan and other regions	Curfew	
April	4	Face mask compulsory in public places		
	19			Face mask compulsory in public places
May	7	Reopening of restaurants, bars, nightclubs, and entertainment venues, only outside Abidjan		
	8	Curfew lifted and public gatherings (200 persons maximum) reauthorized, only outside of Abidjan	Face mask compulsory in public places	
	9		Curfew lifted End of state of emergency	
	11			Curfew adjustments (9 p.m. to 5 a.m.)
	15	Curfew lifted and reopening of restaurants in Abidjan		
June	4			Reopening of restaurants
	7			Curfew adjustments (11 p.m. to 5 a.m.) Intercity travel reauthorized
	30	Reopening of bars, nightclubs, and entertainment venues in Abidjan		Curfew lifted End of state of emergency
July	13	Travel between Abidjan and other regions reauthorized		
August	05			Public gatherings reauthorized Reopening of restaurants, bars, nightclubs, and entertainment venues

The easing of health measures was gradual from May 2020 onwards and began earlier in Côte d'Ivoire and Mali than in Senegal. The curfew was finally lifted on May 9, 2020, in Mali, and on May 15, 2020, in Côte d'Ivoire. Nevertheless, it was not lifted entirely in Senegal until June 30, even though curfew adjustments were introduced on May 11, and intercity travel was again authorized from June 7 onward.

Considering the different measures taken by the governments in response to COVID-19, we identified three periods: (i) *pre-COVID-19* from August 2019 to February 2020, before the implementation of health measures; (ii) *initial emergency response* (March-May 2020), when health measures were most intense (notably with the introduction of a curfew and the restriction of intercity travel); and (iii) the *epidemic management stage* (since June 2020), characterized by the easing of the various measures.

### ATLAS Contingency Plans and COVID-19 Guidance

ATLAS coordination developed contingency plans and COVID-19 guidance as soon as the COVID 19 crisis started. Guidance was shared in March 2020 with all implementing partners focusing on how to protect lay providers and clients; and how HIVST could be an opportunity to maintain access to HIV testing in this context. Personal protective equipment support has also been provided to partners to ensure the protection of peer educators while distributing kits. The guidance was not trying to standardize HIVST distribution during the COVID-19 period and let all implementing partners and peer educators contextualize and adapt their strategies already implemented. Therefore, most activities adaptations described in this article came from the initiative of implementing partners within the frame of ATLAS guidance.

## Results

### HIVST Distribution

Between August 2019 and December 2020, 151,066 HIVST kits were distributed by the ATLAS project among key populations only: 105,788 (70%) through the FSW channel; 40,141 (27%) through the MSM channel; and 5,137 (3%) through the PWUD channel. According to the program data, Côte d'Ivoire accounts for approximately half of all HIVST kits distributed (75,533, 50%), Mali accounts for one-third (54,946, 36%), and Senegal accounts for one-sixth (20,587, 14%).

**Figure 1:**
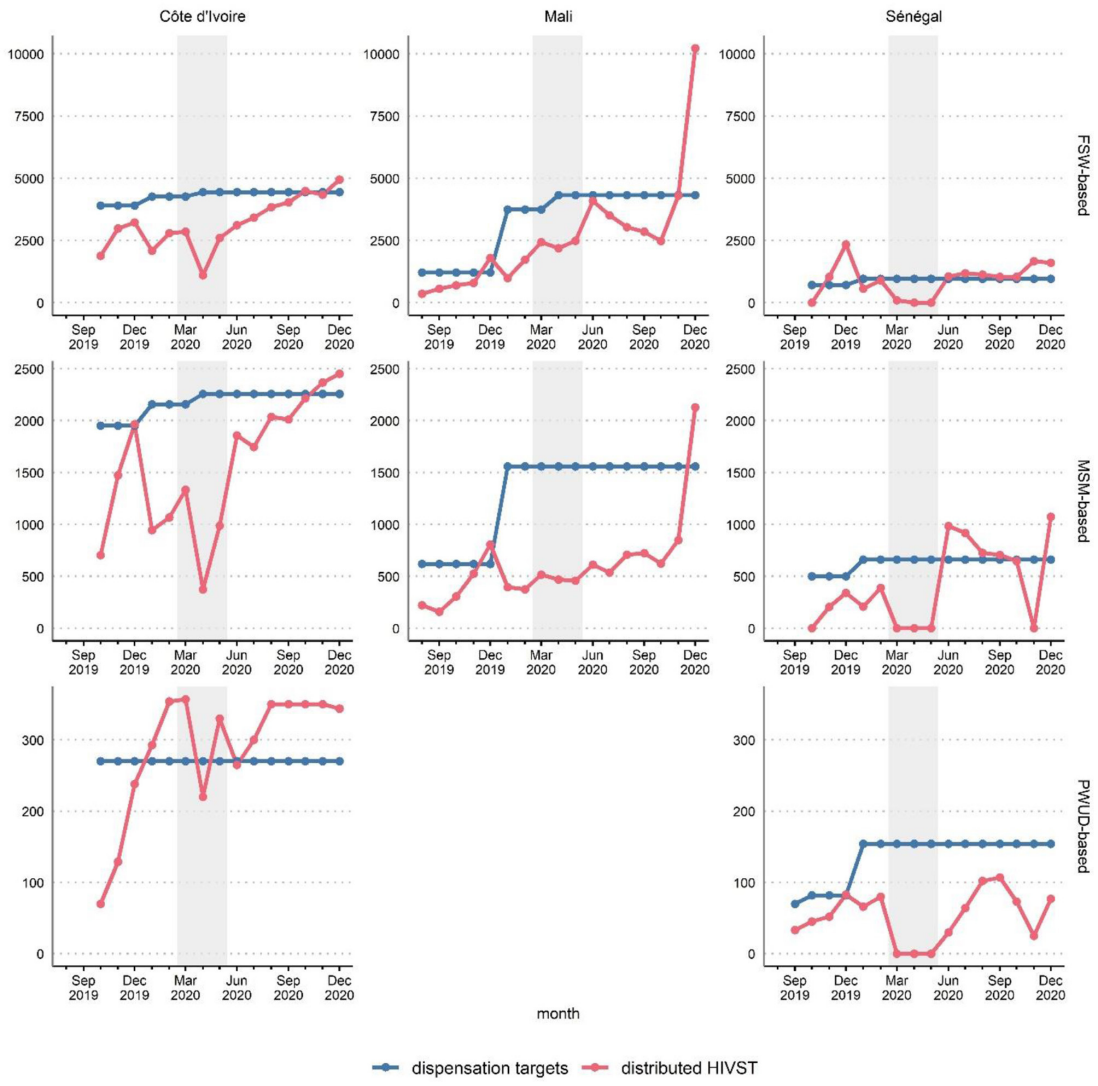
Distribution targets and HIVST distributed during outreach by month, country and delivery channel (FSW-based, MSM-based, or PWUD-based), ATLAS program (August 2019-December 2020). The shaded area corresponds to the emergency COVID-19 response phase (March-May 2020). FSW, female sex workers; MSM, men having sex with men; PWUD, people who use drugs.

In all three countries, the pre-COVID period saw a gradual increase in activities (Figure 1). For some channels, the month of January was marked by a slight decrease caused by a brief delay in the resumption of activities at the beginning of the new year.

During the initial emergency response to COVID-19 (March-May 2020), the distribution evolution differed by country. Senegal witnessed a total cessation of activities during these 3 months, irrespective of the distribution channel. Côte d'Ivoire saw a significant drop in the number of distributed HIVST kits, particularly in April 2020. Mali saw the stabilization of the number distributed (i.e., cessation of the growth observed pre-COVID).

From June 2020 onward, with the routine management of the COVID-19 pandemic, a catch-up phenomenon was observed: activities resumed in Senegal, and the number of distributed HIVST kits rebounded in Côte d'Ivoire and Mali.

### Size of Outreach Activities

**Figure 2:**
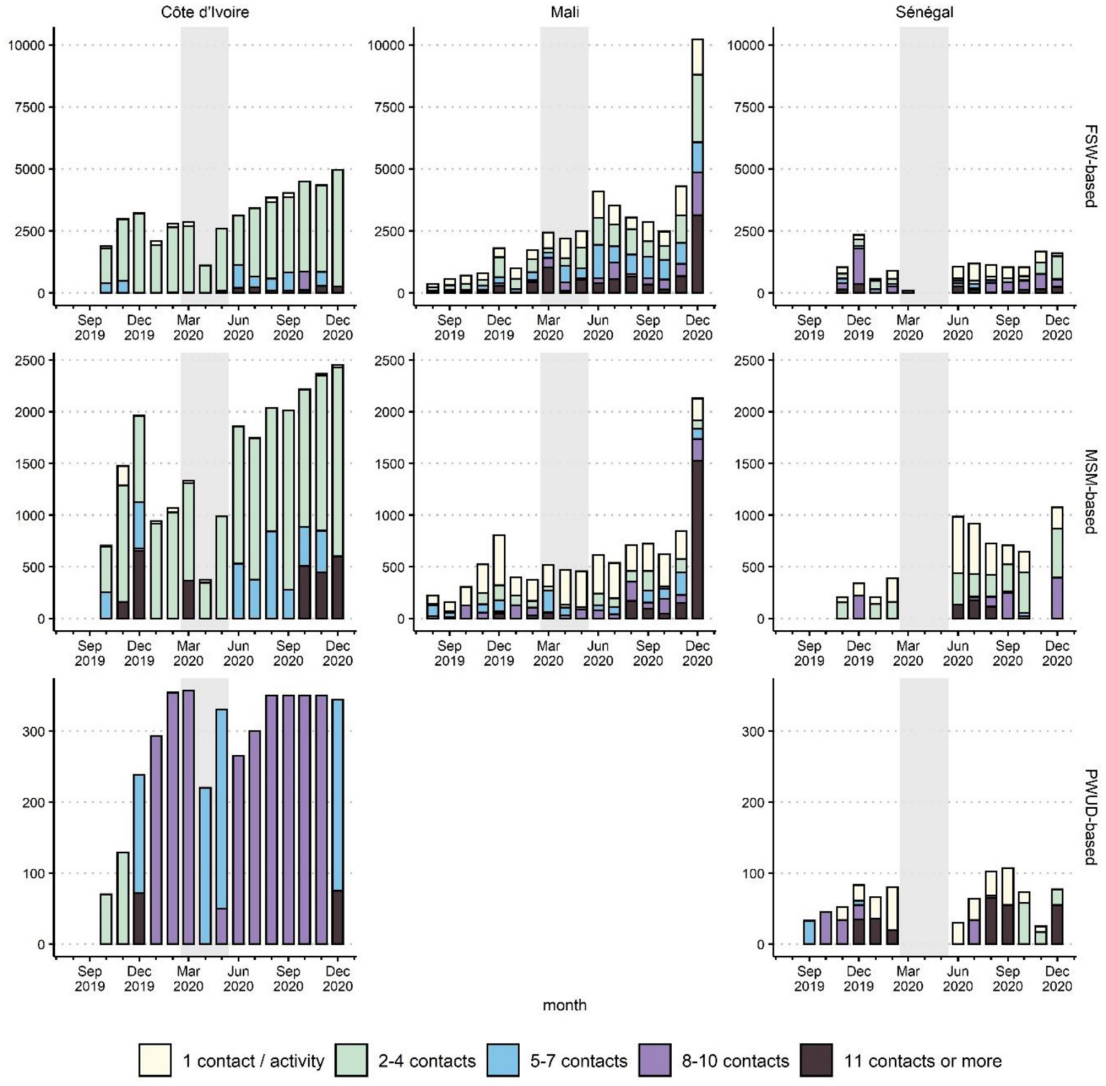
Number of HIVST kits distributed during outreach per average number of primary contacts by activity, per month, country, and delivery channel (FSW-based, MSM-based, or PWUD-based), ATLAS program (August 2019–December 2020). The shaded area corresponds to the emergency COVID-19 response phase (March-May 2020). FSW, female sex workers; MSM, men having sex with men; PWUD, people who use drugs.

Independent of COVID-19, ATLAS outreach activities were heterogeneous across the countries and the key populations, as several intervention models are used (Figure 2 and Supplementary Figures 2, 3).

In Côte d'Ivoire, outreach activities targeting FSW and MSM were usually based on small group talks (2–4 contacts) in public spaces. In addition, social events and parties (11 contacts and more) were organized to reach MSM. In April and May 2020, such social events were suspended. In June 2020 and later, to catch up on distribution, activities for medium-sized groups (5–7 contacts) were organized.

Activities to reach PWUD in Côte d'Ivoire followed a different model: to limit their presence in smoking rooms (sites of drug use) for safety reasons, peer educators intervened during daylight and tried to maximize the number of contacts they made per visit (usually between 8 and 10). During March-May 2020, they maintained the activities but reduced the size of the groups (5–7 contacts per visit).

In Mali, due to the diversity of the implementing partners, several types of activities were conducted to reach FSW and MSM, including home visits, small group activities, and large group activities. As in Côte d'Ivoire, during the emergency phase, large group activities were reduced, and face-to-face activities were prioritized, particularly for the MSM-based channel. This was less the case for the FSW-based channel, as brothels were not closed in all Malian regions.

In Senegal, HIVST implementation used two coexisting distribution models: a model of independent community-based distributors carrying out “one-on-one” activities to reach hidden populations directly and more traditional activities with peer educators working in small groups (e.g., talks, discussion groups, social events). All activities were suspended between March and May 2020. Upon resumption in June 2020, some activities were conducted in larger groups to catch up.

### Age Profile of Primary Contacts

**Figure 3:**
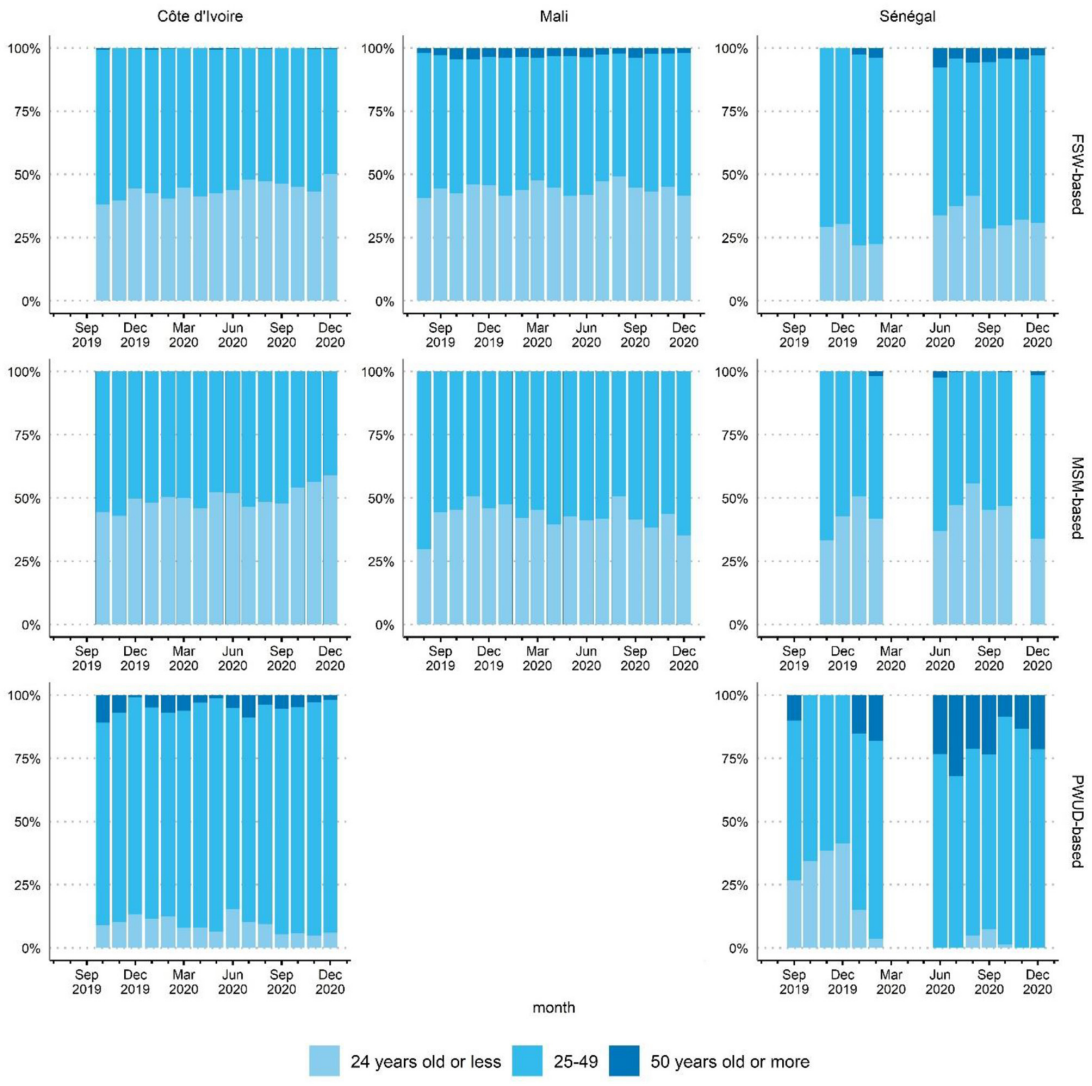
Age profile of primary contacts by month, country and delivery channel (FSW-based, MSM-based or PWUD-based), ATLAS program (August 2019-December 2020). FSW, female sex workers; MSM, men having sex with men; PWUD, people who use drugs.

The age profile of primary contacts was relatively stable over the three reference periods: pre-COVID-19, the emergency phase, and the routine management phase (Figure 3).

### Average Number of HIVST Kits Distributed per Primary Contact

**Figure 4:**
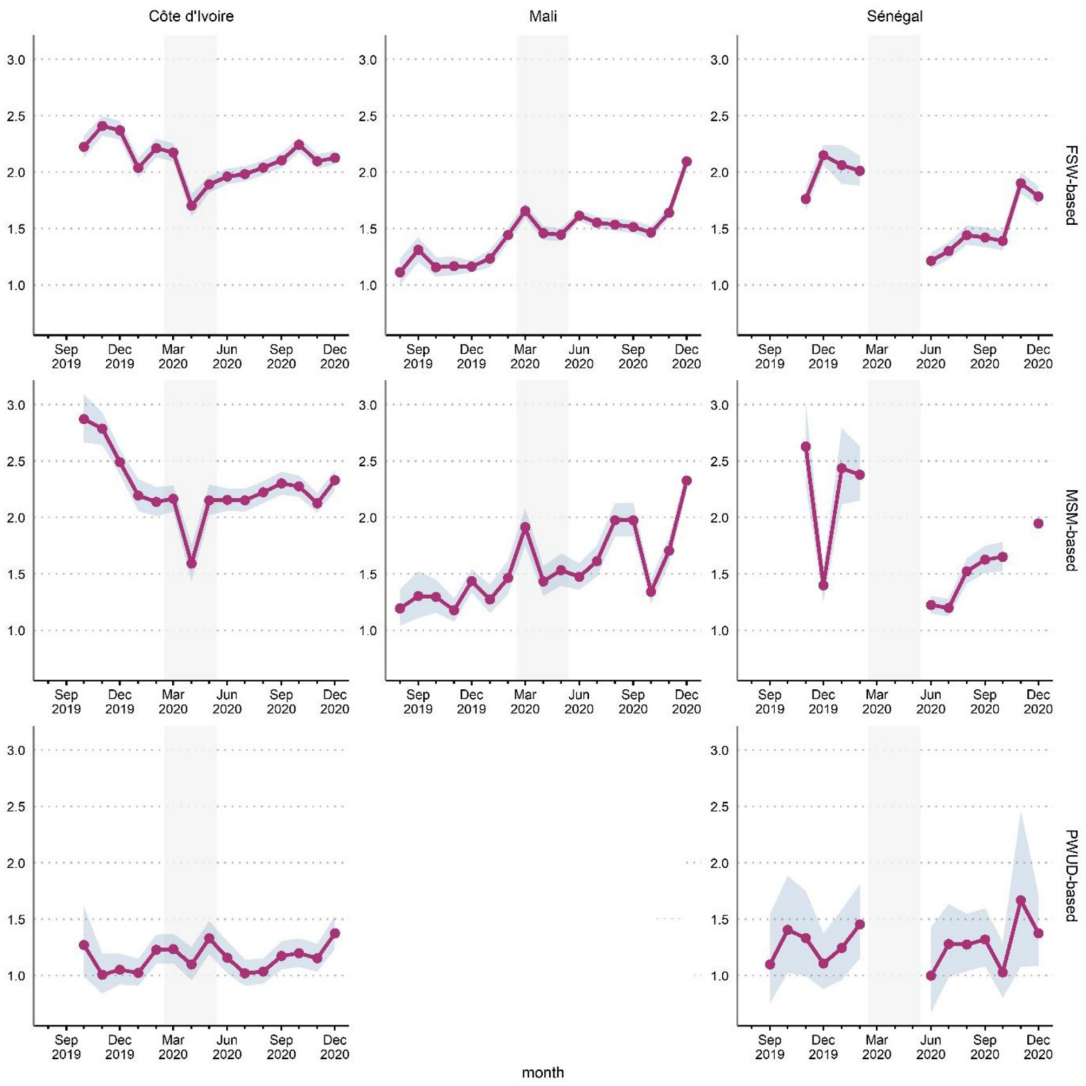
Average number of HIVST kits distributed by primary contacts per month, country and delivery channel (FSW-based, MSM-based or PWUD-based), ATLAS program (August 2019-December 2020). Gray ribbon indicates 95% confidence intervals (Poisson test). The shaded area corresponds to the emergency COVID-19 response phase (March-May 2020). FSW, female sex workers; MSM, men having sex with men; PWUD, people who use drugs.

The average number of HIVST kits distributed per primary contact (Figure 4) is an indirect indicator of secondary distribution.

In Côte d'Ivoire, the closure of bars and restaurants (“maquis”) and the curfew led to a drop in social contacts (in particular for MSM) and a decrease in the number of clients (for FSW), resulting in a decline in the average number of HISVT kits distributed per primary contact. When the curfew was lifted (May 2020), a return to the pre-COVID level in the MSM-based delivery channel was observed, whereas a much slower recovery was observed for the FSW-based channel, with numbers not yet back to the pre-COVID level.

In Mali, this indicator was lower than in the two other countries initially but showed continuous progression over time. The curfew at the end of March 2020, which was extended until early May, led to a drop (slower progress observed). However, there was a recovery and an increase from May onwards in the MSM-based channel and a plateau in the FSW-based channel.

In Senegal, activities restarted in June, with a significant setback compared to the pre-COVID period. Despite a gradual recovery, the average number of HIVST kits distributed per primary contact had not yet reached its pre-crisis level by the end of 2020.

### Adjustments of HIVST Activities to Comply With Governmental Health Measures

The measures taken by governments (Table 1) and the application of hygiene measures led to major changes in HIVST outreach activities between March and May 2020. Qualitative feedback from peer educators (through individual interviews and focus groups) is summarized in Table 2.

**Table 2:** Adaptation of HIVST distribution outreach activities according to peer educators' feedback, 2020, ATLAS program.

**Delivery Channel**	**Côte d'Ivoire**	**Mali**	**Senegal**
FSW-based	• **Adaptation of activities (March-May 2020)** • From public to private spaces • Group size reduction • Night activities moved to daytime • Rapid tests converted into assisted HIVST • Appointment by phone/WhatsApp • Hygiene measures^*^	**Adaptation of some activities (March-May 2020, region dependent)** • From public to private spaces • Group size reduction • Night activities moved to daytime • Rapid tests converted • Into assisted HIVST • Appointment by phone/WhatsApp • Hygiene measures^*^	• **Suspension of outreach activities (March–May 2020)** • **Resumption of activities (June 2020)** • Prioritization of face-to-face activities • Less HIVST distributed per contact • Appointment by phone/WhatsApp • Hygiene measures^*^
MSM-based	• **Adaptation of activities (March-May 2020)** • From public to private spaces • Group size reduction • Night activities moved to daytime • Rapid tests converted into assisted HIVST • Increased use of social networks • Hygiene measures^*^	• **Adaptation of some activities (March-May 2020, region dependent)** • From public to private spaces • Group size reduction • Night activities moved to daytime • Rapid tests converted into assisted HIVST • Increased use of social networks • Hygiene measures^*^	• **Suspension of outreach activities (March-May 2020)** • **Resumption of activities (June 2020)** • Prioritization of face-to-face activities • Less HIVST distributed per contact • Appointment by phone/WhatsApp • Increased use of social networks • Hygiene measures^*^
PWUD-based	• **Adaptation of activities (March-May 2020)** • Unchanged intervention sites (smoking sites) • Group size reduction • Unchanged timing (daytime) • Rapid tests converted into assisted HIVST • No use of social networks • Hygiene measures^*^		• **Suspension of outreach activities (March-May 2020)** • Referral to a dedicated clinic (Dakar) • **Resumption of activities (June 2020)** • Prioritization of face-to-face activities • Less HIVST distributed per contact • No use of social networks • Hygiene measures^*^

* *Hygiene measures: awareness of COVID-19, wearing a mask (distributor), hydroalcoholic gel (distributors + primary contacts), physical distancing (sometimes difficult)*.

*FSW, female sex workers; MSM, men having sex with men; PWUD, people who use drugs*.

In Côte d'Ivoire, peer educators made several adjustments for activities targeting FSW and MSM: relocation from public (bars, venues, brothels, etc.) to private areas (home, discreet places, etc.); group size reduction with prioritization of face-to-face talks when possible. Peer educators reported similar adjustments in Mali, with variations by region depending on how closely local populations have followed governmental health measures.

FSW peer educator, focus group, Mali: “*There have been many changes in our work. Before, people used to come to the maquis* [local restaurants]*, but after the maquis were closed down and the FSW were obliged to go and take rented flats, we used to go to these homes to give talks, and we were obliged to do so for as long as they could give us. We don't go out into the field at night to go to work anymore*.”

All outreach activities conducted at night were stopped by the different curfews and were rescheduled for the daytime.

Social networks (Facebook, Messenger, WhatsApp), commonly used by MSM, were increasingly used by MSM peer educators during March-May 2020 to maintain contact with their peers, promote HIV prevention and testing and organize face-to-face or small group meetings.

MSM peer educator, focus group, Côte d'Ivoire: “*In the COVID-19 period, since we couldn't really meet I did everything online, that's it; I was raising awareness online. When it comes to dispensing self-tests now, I move around, we meet up and then I give.”*

MSM peer educator, focus group, Côte d'Ivoire: “*I created a Facebook group “les branchés de* [small town in Côte d'Ivoire]”*. I created a second group “les branchés de* [other small town]”*, and I publish photos, videos, images in a trendy way; we know each other and others have asked to join. And it's like I've broadened my thing a bit and now I'm going out there to go door to door.”* [‘branchés' is a term used by MSM to refer to themselves.]

FSW peer educators used social networks mainly to make appointments or keep in touch with their peers. Unlike MSM, social networks were not used to expand the peer network.

FSW peer educator, focus group, Mali: “*If we didn't know their homes, we called them and looked for their homes.”*

In Côte d'Ivoire, activities with PWUD have been maintained within the smoking rooms. However, the number of visits and the number of contacts per visit have been reduced.

PWUD peer educator, focus group, Côte d'Ivoire: “*At the beginning, we had seven visits* [per week]*, but when COVID arrived, we went down to five visits*.”

PWUD peer educator, focus group, Côte d'Ivoire: “*We had to avoid being too in contact with the DU* [drug users] *because they are glued, they like contact! That is to say that if he is not with you, he is not at ease*.”

PWUD peer educator, focus group, Côte d'Ivoire: “We divided up, we took them in small groups.”

In Senegal, activities were suspended from March to May 2020.

MSM peer educator, focus group, Senegal: “*The context of COVID has impacted the work because we have gone for months without distributing HIVST, and this impacts the achievement of our distribution objectives.”*

### Rapid Application of Hygiene Measures by Peer Educators

In all three countries, the application of hygiene measures was welcomed by peer educators as offering protection from COVID-19.

MSM peer educator, individual interview, Côte d'Ivoire: “*We are not afraid anymore because we have the means to protect ourselves; there are the gels, there is everything and then we always continue to respect the barrier measures; even if it is not 100%, we respect them all the same*.”

Maintaining Physical Distancing Was the Most Difficult Measure to Implement

FSW peer educator, individual interview Côte d'Ivoire: “*If I am one meter away from the peers and I speak I am obliged to get closer, especially in a bar/maquis, to remain discreet, but I always wear the face mask.”*

Some peer educators mentioned the difficulty of not having face masks to distribute to users. For example, some peer educators decided to give them a face mask from their personal dotation when some users did not have a face mask. This meant that the peer educator could not change their face mask as often as recommended.

FSW peer educator, focus group, Mali: “*I think there can be a problem if you are protected and not me, because if you are protected and the rest of us are not, we can be exposed when you come to do the demonstration. So if we are all protected, there is no problem.”*

PWUD peer educator, focus group, Côte d'Ivoire: “*So when you arrive on the sites, it's when DU* [drug users] *asks you “Can I have a face mask too?” That's when you give them a face mask, your face mask that is on you that you give them to wear* [i.e. the peer educator gave a mask from his personal dotation, not the mask he was currently wearing at the time]. *Otherwise, we don't have face masks to share.”*

Within a few weeks, hygiene measures were routinely integrated.

PWUD peer educator, focus group, Côte d'Ivoire: “*Everyone is now used to wearing masks*.”

### Assisted HIVST: A Safe Replacement for Rapid HIV Testing When Physical Distancing Is Needed

Before the COVID-19 crisis, peer educators proposed both conventional rapid HIV testing and HIVST. In March-April, the lack of personal protective equipment, in particular face masks and hydroalcoholic gels, made the application of hygiene measures difficult. Physical distancing was favored during activities. Due to the challenge of safely performing rapid testing in such a context, some peer educators proposed assisted HIVST as a replacement for rapid testing for those who agreed to be tested onsite.

MSM peer educator, individual interview, Côte d'Ivoire: “*Since March when we were talking about distancing, it was a bit difficult even to do the classic tests. We took a lot of advantage of the self-tests because at least you can offer them.”*

MSM peer educator, focus group, Mali: “*Our work doesn't allow us to respect safety measures; it's a bit difficult. So I myself from the beginning of the coronavirus until recently, most of my screening is done through self-testing. I give it to you, and I explain it to you, so you do your test, even if it's assisted, you do it, and when you're done doing it, we'll do what needs to be done.”*

PWUD peer educator, focus group, Côte d'Ivoire: “*HIVST helped to maintain the link during the crisis.”*

### HIVST Activities: An Opportunity for COVID-19 Awareness-Raising

Initially, peer educators reported that some key population members perceived hygiene measures as a form of discrimination. Peer educators were gradually able to provide information about COVID-19 and thus promote the importance of these measures. This awareness-raising complemented the governmental messages about COVID-19.

PWUD peer educator, focus group, Côte d'Ivoire: “*We tried to get them to understand that they should try to separate a little, try to loosen up a little. It was difficult; we had to rehearse*.”

PWUD peer educator, focus group, Côte d'Ivoire: “*They finally understood that it wasn't because of their status but because of COVID.”*

### Gradual Return to Normal With the Maintenance of Hygiene Measures

When activities resumed in June 2020, they were re-adapted: face-to-face activities were prioritized when possible, and activities were moved to private areas and the daytime. It was also reported that instructions were given to distribute only one HIVST kit per contact.

FSW peer educator, focus group, Senegal: “*Before COVID, we used to go out at night to distribute to bars and restaurants. But with the pandemic and the restrictive measures taken on that occasion, we were obliged to change our strategy and give priority to home visits*.”

MSM peer educator, focus group, Senegal: “*In November and December, we were told that up to 3 HIVST kits could be distributed per MSM. But after the resumption of activities in the post-COVID period, between July and August, they came back and told us as an independent distributor to distribute 1 HIVST kit per person from now on*.”

With the easing of public health measures and the routinization of COVID management, activities have gradually returned to as they were before the crisis: held in public places, with larger groups, and sometimes in the evening.

FSW peer educator, focus group, Mali: “*Activities have resumed almost as before. Places have reopened, and people are no longer picked up from their homes but rather from their usual places*.”

Some peer educators suggested maintaining such preventive measures even after the COVID-19 pandemic to prevent other communicable diseases, such as tuberculosis.

FSW peer educator, individual interview, Mali: “*For me, there are changes that we have to maintain because even after COVID-19 there are other communicable diseases; these are the means of protection that we have put in place*.”

## Discussion

The pre-COVID-19 period allowed for a gradual distribution of HIVST in the three countries, with many activities carried out in large groups (5 or more contacts), varying according to the country and the type of targeted key population. During the initial emergency response period (March-May 2020), activities were severely disrupted with a total suspension in Senegal, a significant drop in Côte d'Ivoire, and a less pronounced drop in Mali. Priority was given to activities conducted in small groups (4 contacts or less). Secondary distribution (measured indirectly by the average number of HIVST distributed per primary contact) was also negatively affected. To ensure continuity of activities, peer educators in charge of HIVST distribution showed resilience and adapted by moving from public to private areas, reducing group size, shifting night-time activities to daytime, increasing the use of social networks, integrating hygiene measures, and promoting assisted HIVST as an alternative to traditional rapid testing.

With routine management of the pandemic from June 2020 onwards, a catch-up phenomenon was observed: activities resumed in Senegal, new distribution sites were established, the number of HIVST distributed rebounded, the activities of larger groups resumed, and the average number of HIVST distributed per primary contact rebounded.

Using quantitative and qualitative data from activity reports, individual interviews, and focus groups, our main findings highlight the significant but heterogeneous impacts of COVID-19 disruptions on ATLAS project activities and how peer educators and implementing partners have been able to adapt in such context and showed resilience. The flexibility of HIV self-testing strategies allowed the maintenance of access to HIV testing services for key populations while ensuring hygiene measures.

Our results need to be interpreted in light of some limitations. Unlike survey data, which are usually collected at an individual level, monthly reports are aggregated by site and delivery channel. In addition, though the number of distributed HIVST kits (main indicator) is reported fairly precisely, less attention is given to the number of primary contacts, the number of activities, or the type of activities. Only outreach activities have been considered in this analysis, and it would be relevant to explore the impact on facility-based activities as well. During the crisis, individual interviews were conducted by phone with the primary objective of documenting the challenges faced by program implementers, limiting the depth of these interviews. Finally, the data being collected on behalf of Solthis, the body to which CSOs report their activities, may be subject to response and desirability bias.

However, developing a dedicated survey would have required several months (development, funding, authorizations) before being implemented, and it would not have been possible to observe changes and adaptations of activities during the initial emergency response phase. In that sense, using routinely collected monitoring data for secondary analysis provides valuable information.

Worldwide, the COVID-19 pandemic has impacted all health sectors, including global HIV strategies (24). Emergency public health measures have limited populations' freedom of movement, resulting in lower access to essential HIV prevention, testing, and treatment services (25–27). West Africa has been no exception; the governmental health measures in Côte d'Ivoire, Mali, and Senegal have impacted the daily lives of key populations. For ATLAS, HIVST distribution was disrupted, and secondary distribution was limited. Similarly, there were program-level effects, such as the delayed opening of certain distribution sites (Supplementary Figure 4).

However, there is no evidence if risky behaviors may have increased or decreased among key populations during the period where governmental restrictions were in place. For example, the closure of bars/restaurants and curfews may have reduced the number of clients of FSW (reducing exposure to HIV), but condom negotiation may have been more difficult (increasing exposure to HIV). If our results show that HIVST offer has been reduced due to the adaptation of activities, we have no feedback from peer educators that HIVST demand decreased, except probably for secondary distribution (as it was more challenging to redistribute HIVST kits in such a context).

ATLAS's implementing partners had to adapt their operational procedures to ensure service continuity in an emergency context where COVID-19 was not well-known and the discourse on hygiene measures varied from country to country.

In Senegal, where governmental measures were scrupulously followed, local partners decided to suspend activities for two main reasons. First, Senegalese community-based organizations are extremely cautious in a country where stigma toward key populations is high and media scandals frequent. Second, there were financial issues during this period. ATLAS's HIVST outreach distribution is integrated within traditional testing activities funded by other donors. The principal ATLAS community-based partner in Senegal for FSW and MSM was withdrawn from a Global Fund grant in January 2020, resulting in a suspension of certain activities. Nevertheless, HIVST distribution continued through the independent community distributors, and CSO-based activities resumed in June 2020.

In Côte d'Ivoire, where governmental measures were globally respected, HIVST distribution was maintained with considerable adaptation by peer educators.

In Mali, where governmental measures were weaker, and adherence varied according to region, HIVST distribution was less impacted.

From June onward, the easing of public health measures allowed a relative return to normal. During this process of routinization, hygiene measures and COVID-19 awareness-raising were maintained in the field by HIVST distributors, ensuring the continuity of testing activities to optimize key populations' monitoring and management (28).

From our main results, different lessons can be drawn from the ATLAS project activity reports on the provision of HIVST in the context of the COVID-19 health crisis. Peer educators and key populations have been adaptive and resilient in deploying strategies to ensure continuity of distribution activities while integrating health constraints (22, 29). These adaptations made it possible to maintain access to HIV testing while respecting the barrier measures. HIVST has also helped to maintain access to testing, and its delivery is flexible enough to adapt to different contexts (30, 31).

## Conclusion

Although imperfect, program data provide valuable information to describe changes in the implementation of HIVST outreach activities over time. The impact of the COVID-19 pandemic on HIVST distribution among key populations was visible in the monthly activity reports. Activities and secondary distribution were disrupted. Focus groups and individual interviews allowed documentation of the adaptations made by peer educators, with variations across countries and populations: relocating activities from public to private areas, reducing group sizes, moving night activities to the daytime, increasing the use of social networks, integrating hygiene measures, and promoting assisted HIVST as an alternative to conventional rapid testing… These adaptations demonstrate the resilience and learning capacities of peer educators and key populations. However, the uncertain evolution of the COVID-19 epidemic in 2021, with the possibility of new waves, could lead to additional impacts on activities.

## Data Availability Statement

The raw data supporting the conclusions of this article will be made available by the authors, without undue reservation.

## Author Contributions

GC collected qualitative data. PD, KH, BDia, and BDie managed quantitative data collection. AK, GC, PD, AV, and JL conceived and designed the analysis. GC did the qualitative analysis. AK did the statistical analysis. AK, GC, and JL wrote the first draft of the manuscript. All authors contributed to the interpretation and presentation of the findings and approved the final version of the manuscript for submission.

## Conflict of Interest

The authors declare that the research was conducted in the absence of any commercial or financial relationships that could be construed as a potential conflict of interest.
